# The Effect of Mechanical Stretch on Myotube Growth Suppression by Colon-26 Tumor-Derived Factors

**DOI:** 10.3389/fcell.2021.690452

**Published:** 2021-07-30

**Authors:** Jessica L. Halle, Brittany R. Counts-Franch, Rose M. Prince, James A. Carson

**Affiliations:** Integrative Muscle Biology Laboratory, Division of Rehabilitation Sciences, College of Health Professions, University of Tennessee Health Science Center, Memphis, TN, United States

**Keywords:** cancer cachexia, skeletal muscle (myotubes), myosin heavy chain, myogenic differentiation, passive stretch

## Abstract

Preclinical models and *in vitro* experiments have provided valuable insight into the regulation of cancer-induced muscle wasting. Colon-26 (C26) tumor cells induce cachexia in mice, and conditioned media (CM) from these cells promotes myotube atrophy and catabolic signaling. While mechanical stimuli can prevent some effects of tumor-derived factors on myotubes, the impact of mechanical signaling on tumor-derived factor regulation of myosin heavy chain (MyHC) expression is not well understood. Therefore, we examined the effects of stretch-induced mechanical signaling on C2C12 myotube growth and MyHC expression after C26 CM exposure. C26 CM was administered to myotubes on day 5 of differentiation for 48 h. During the last 4 or 24 h of C26 CM exposure, 5% static uniaxial stretch was administered. C26 CM suppressed myotube growth and MyHC protein and mRNA expression. Stretch for 24 h increased myotube size and prevented the C26 CM suppression of MyHC-Fast protein expression. Stretch did not change suppressed MyHC mRNA expression. Stretch for 24 h reduced Atrogin-1/MAFbx, MuRF-1, and LC3B II/I ratio and increased integrin β1D protein expression and the myogenin-to-MyoD protein ratio. Stretch in the last 4 h of CM increased ERK1/2 phosphorylation but did not alter the CM induction of STAT3 or p38 phosphorylation. These results provide evidence that in myotubes pre-incubated with CM, the induction of mechanical signaling can still provide a growth stimulus and preserve MyHC-Fast protein expression independent of changes in mRNA expression.

## Introduction

Cancer-induced skeletal muscle wasting, or cancer cachexia, is multifactorial and involves a complex interplay between host- and tumor-derived factors resulting in disrupted skeletal muscle function and metabolism (Jackman et al., 2017; Argiles et al., 2019). Posture, breathing, and daily activities require functional skeletal muscle. While dynamic exercise promotes metabolic and functional adaptations, mechanical stimulation of muscle involving passive movement (i.e., stretch) can produce important functional adaptations (Carson et al., 1995; Carson, 1997; De Deyne, 2001; Zöllner et al., 2012; Schiaffino et al., 2013; Hardee et al., 2016). Critical gaps remain in our knowledge of how tumor-derived cachectic factors influence mechanotransduction in cachectic muscle. Furthermore, it is not well understood if mechanical stimulation can induce growth or prevent further atrophy in muscle that has been exposed to cachectic tumor-derived factors. While our mechanistic understanding of the drivers of cancer cachexia has advanced significantly, it is also critical to investigate the constraints that cachexia places on intrinsic muscle properties, such as mechanical regulation. Furthermore, a better understanding of how increased mechanotransduction may elicit beneficial effects on cancer-induced muscle wasting is necessary.

Stretching cultured myotubes can increase the protein synthesis rate and size. Furthermore, stretch activates mechanical sensitive signaling involving ERK1/2, p38 MAPK, and JNK signaling while decreasing muscle-specific proteolytic factors (Kumar et al., 2002; Pereira et al., 2011; Moustogiannis et al., 2020). Lewis lung carcinoma (LLC) cachectic factors can impair stretch-induced protein synthesis (Gao and Carson, 2016). However, the effects of stretch on markers of autophagy flux and protein degradation such as the E3 ubiquitin ligases Atrogin-1/MAFbx and MuRF-1 in myotubes exposed to cachectic factors is less understood. Different tumor types can differentially release factors that can target myotube and skeletal muscle dysfunction and wasting (Freire et al., 2020). While LLC factors can disrupt myotube stretch-induced signaling (Gao and Carson, 2016), it is not well established if cachectic colon-26 (C26) tumor-derived factors can inhibit mechanical signaling in myotubes. Transmembrane integrin proteins can transmit mechanical signaling into the cell. Skeletal muscle β1D integrin isoform has an established role in myoblast differentiation and muscle fiber growth (van der Flier et al., 1995; Carson and Wei, 2000). The activation of myogenic transcription factors, including myogenin and MyoD, contributes to stretch-induced hypertrophy (Carson and Booth, 1998). Dynamic stretch of myotubes during treatment with C26 tumor-derived factors can increase myotube size, promote myoblast differentiation, and improve myogenesis markers (i.e., myogenin and MyoD) related to myotube fusion (Baccam et al., 2019). Stretch of myotubes is also associated with myokine release and an improved follistatin/activin ratio (Baccam et al., 2019). The role of myosin heavy chain (MyHC) as a contractile protein is fundamental for cross-bridge formation and skeletal muscle function (Schiaffino and Reggiani, 1994; Toth et al., 2013). MyHC loss occurs with C26 cachexia (Diffee et al., 2002; Yamada et al., 2020). Although myotube size measured by diameter is often increased with stretch (Vandenburgh and Karlisch, 1989; Gao and Carson, 2016), conflicting results have been reported relating to stretch and MyHC expression. Studies often examine MyHC as a marker of myotube differentiation in stretched myoblasts (Wang et al., 2020). The interaction of passive stretch and C26 cachectic factors on myogenesis and MyHC at late stages of differentiation could offer valuable insight into mechanisms mediating skeletal muscle repair and regeneration.

Colon-26 carcinoma *in vivo* is associated with a progressive loss of skeletal muscle mass and function mediated by increased circulating pro-inflammatory cytokines and the activation of muscle-specific E3 ligases (Aulino et al., 2010; Bonetto et al., 2011, 2016; Halle et al., 2019). Preclinical cachexia models have provided valuable insight into how host- and tumor-derived factors regulate muscle mass. *In vitro* culture models have provided a further mechanistic understanding of tumor-derived factors’ potential direct effects on myotubes using tumor cell conditioned media (CM) (Jackman et al., 2017). C26 CM induces myotube atrophy and intrinsic signaling involving JAK/STAT, p38 MAPK, ERK1/2 activation, and muscle-specific E3 ubiquitin ligase expression (Seto et al., 2015; Jackman et al., 2017; Liu et al., 2019). While exercise can prevent skeletal muscle atrophy and dysfunction in C26 tumor-bearing mice (Pigna et al., 2016; Ballaro et al., 2019), it is not fully established if exercise-induced systemic alterations or intrinsic muscle signaling involving contraction or stretch are responsible for these effects. Dynamic stretch occurring with the administration of C26-derived catabolic factors for 48 h maintains the ability to promote increased myotube diameter (Baccam et al., 2019). However, the stretch response after the initiation of C26-induced myotube catabolism and whether mechanical signaling can generate a growth stimulus in an already catabolic state warrants further investigation. Furthermore, the interaction of mechanical signaling and C26 CM in a high serum, growth-promoting state is also less understood. While MyHC expression is critical for myotube differentiation and growth, there is a limited understanding of how stretch-induced mechanical signaling impacts the regulation of myotube MyHC expression by tumor-derived factors. Therefore, we examined the effects of mechanical signaling induced by both acute and chronic passive stretch on C2C12 myotube growth and MyHC expression when previously exposed to C26 CM. C26 CM was administered to C2C12 myotubes at day 5 of differentiation for 48 h. During the last 4 or 24 h of C26 CM exposure, a subset of samples were administered 5% static uniaxial stretch.

## Materials and Methods

### Cell Culture

All cells were purchased through ATCC (Manassas, VA, United States) and used within the first 15 passages. Murine C2C12 myoblasts (CRL-1772), C26 adenocarcinoma (CRL-2638), or EL4 lymphoma (TIB-39) cells were maintained at 37°C, 5% CO_2_ in growth media (GM): Dulbecco’s Modified Eagle Media (DMEM; #11995-065; Gibco, Grand Island, NY, United States) supplemented with 10% fetal bovine serum (FBS), 50 U/ml of penicillin, and 50 μg/ml of streptomycin.

### C2C12 Myotube Differentiation

C2C12 myoblasts were seeded on type I collagen-coated polystyrene plastic or flexible silastic Uniflex^®^ membranes (Flexcell International, Burlington, NC, United States) at a density of 8.0 × 10^4^ cells per well (six-well plate) in GM: DMEM (#11995-065; Gibco) supplemented with 10% FBS, 50 U/ml of penicillin, and 50 μg/ml of streptomycin. To induce myoblast differentiation, cells were rinsed with phosphate-buffered saline (PBS) and switched to differentiation media (DM): DMEM supplemented with 2% heat-inactivated horse serum, 50 U/ml of penicillin, and 50 μg/ml of streptomycin to form myotubes. Media was replenished every 48 h, and experiments were performed starting at day 5 of differentiation when multinucleated contractile myotubes are present.

### Conditioned Media Collection

Colon-26 cells, a murine colon adenocarcinoma that promotes cachexia in mice, or EL4 lymphoma cells that do not promote cachexia in mice (Bonetto et al., 2016; Zhang et al., 2017), were cultured as described above. Conditioned media (CM) consists of consists of secreted factors from tumor cells and has been described previously (Kandarian et al., 2018). Briefly, 2 × 10^6^ cells were seeded in 100-mm tissue culture-treated plates in GM. Tumor cell CM was collected at ∼90% confluence 48 h post cell seeding and spun down at 3,000 rpm for 5 min to remove cell debris. Cells on the plate were pelleted and counted via trypan blue exclusion test to ensure an equivalent number of cells on the plate, with a final density averaging 7.0–9.0 × 10^6^ cells per culture dish. CM was stored for one-time use in aliquots at −20°C, used within 2 months, and then thawed in a warm water bath at the time of the experiment. GM with no cells was used as a media control.

### Treatment of Myotubes With Conditioned Media

At the time of the experiment, GM control or C26 CM is diluted with 50% serum-free DMEM for a final serum concentration of 5% FBS; 50% CM was chosen, as it has been shown to produce significant myotube atrophy (Pin et al., 2018; Zhong et al., 2019). It should be noted that myotubes were differentiated up to day 5 in 2% horse serum; thus upon CM addition, myotubes were switched to a higher serum environment, which can induce myotube hypertrophy (von Walden et al., 2016). Therefore, in the figures and results, EL4 and C26 CM are referred to as EL4 + GM or C26 + GM. A mixture of myoblasts and myotubes at day 5 of differentiation was rinsed with PBS and then switched to GM media control or C26 CM (C26 + GM) for 48 h (days 5–7 of differentiation). CM was replenished after 24 h. In a separate experiment, C2C12 myoblasts were grown and differentiated on type I collagen-coated rigid polystyrene plates to examine the effects of the cachectic tumor cell CM on a commonly utilized substratum. Briefly, differentiated myotubes at day 5 were treated with 50% GM, C26, or EL4 CM (EL4 + GM) for 48 h. EL4 CM was used to determine if effects on myotube growth are specific to cachectic tumor-derived factor media.

### Myotube Uniaxial Stretch With Conditioned Media

Static stretch was administered (FX-6000 Tension System, Flexcell International Corporation) as previously described (Hornberger et al., 2005; Baccam et al., 2019) with the following modifications. Cells were subjected to a static 5% uniaxial stretch in the last 4 or 24 h of CM treatment (as described above). Control cells (0% stretch) were grown under identical conditions but left unstretched and placed beside the incubator’s baseplate. For administration of stretch, culture plates were removed from the incubator, rapidly placed onto the stretch device’s baseplate, and then placed back in the incubator. The Flexcell Arctangle^®^ loading station was used for all experiments, which consists of six rigid posts covered in a thin layer of silicone lubricant, centered beneath the six-well Uniflex plate. Applied vacuum pressure deforms the membrane across the loading post in a single direction (e.g., north and south poles), creating a uniaxial stretch (Figure 3B). A custom ramp protocol, described here, was designed to ensure cell adherence to the membrane while vacuum pressure is applied: the Flexcell tension system was set to increase vacuum pressure gradually, and thus % elongation in increments of 1% every 2 s, up to 5%, which is then maintained for the duration of the experiment (e.g., 24 h), followed by a ramp-down to 0% at the conclusion of the experiment.

### Protein Synthesis Measurement

Protein synthesis was measured in myotubes treated with CM grown on type I collagen-coated polystyrene plates using the surface sensing of translation (SUnSET) method as previously described (Goodman et al., 2011). Puromycin (#540411; Sigma-Aldrich, St. Louis, MO, United States) was added to culture media 30 min prior to protein harvest at a final concentration of 1 μM per well. The amount of puromycin incorporated into newly synthesized protein was determined by Western blotting.

### Myotube Diameter

Myotube diameter was quantified as previously described (Gao and Carson, 2016) with the following modifications. C2C12 myotube diameter was quantified using ImageJ software (National Institutes of Health, Bethesda, MD, United States). Digital images were captured at ×20 objective brightfield. Five non-overlapping images were captured within each well, and three images were randomly chosen for the analysis. The analysis used unmodified tiff images accessed in NIH ImageJ software. A blinded investigator randomly took diameter measurements of six myotubes per image based on preset inclusion/exclusion criteria: elongated structure with distinct membrane outlines, little to no cellular debris, and no branching points. The average diameter per myotube was calculated as the mean of eight measurements taken along the myotube length. For each condition, *n* = 162–378 myotubes were analyzed.

### Western Blotting

Western blotting analysis was performed as previously described (Gao and Carson, 2016). Briefly, cells were scraped into ice-cold radioimmunoprecipitation assay (RIPA) buffer [25 nM of Tris HCl at pH 7.6, 150 nM of NaCl, 1% NP-40, 1% sodium deoxycholate, and 0.1% sodium dodecyl sulfate (SDS)] (#89900; Thermo Fisher Scientific, Waltham, MA, United States) and 1% Halt protease and phosphatase inhibitor cocktail [1 mM of EDTA, 5 mM of NaF, 1 nM of NaVO_4_, and 1 mM of glycerophosphate] (#78440; Thermo Fisher Scientific). Cell lysates were centrifuged at 14,000 rpm for 15 min at 4°C, and supernatants were collected. Protein concentrations were determined using the Bradford method. Homogenates were fractionated on SDS–polyacrylamide gels and transferred to polyvinylidene difluoride (PVDF) membrane. Membranes were blocked for 1 h in 5% non-fat milk–TBST. After blocking, primary antibodies for phosphorylated STAT3 (Y705) 1:1,000, total STAT3 1:2,000, phosphorylated p38 (T180/Y182) 1:1,000, total p38 1:2,000, phosphorylated ERK1/2 (T202/Y204) 1:1,000, total ERK1/2 1:2,000, phosphorylated rpS6 (S240/244) 1:1,000, and total rpS6 1:2,000, phosphorylated Akt (S473) 1:1,000, total Akt 1:2,000, phosphorylated 4E-BP1 (T37/46) 1:1,000, total 4E-BP1 1:2,000, phosphorylated SAPK/JNK (T183/Y185) 1:1,000, total SAPK/JNK 1:2,000 (Cell Signaling Technology, Danvers, MA, United States), Atrogin-1/MAFbx, MuRF-1 1:1,000 (ECM Biosciences, Versailles, KY, United States), and MyHC-Fast (protein stain for fast type II fibers; corresponding genes: MyH1 and MyH2) and MyHC-Slow 1:4,000 (protein stain for slow type I fibers; corresponding gene MyH7), serum response factor (SRF) 1:500, myogenin 1:500, and MyoD 1:500 (Santa Cruz Biotechnology, Dallas, TX, United States), integrin β1D 1:1,000, and puromycin 1:2,000 (Millipore, Billerica, MA, United States) were incubated overnight at 4°C in 5% non-fat milk–TBST. Secondary anti-rabbit or anti-mouse IgG horseradish peroxidase (HRP)-linked (Cell Signaling Technology) antibodies were incubated at 1:4,000 dilution for 1 h at room temperature in 5% non-fat milk–TBST. Enhanced chemiluminescence (Prometheus Pro-Signal Femto, #20-302; Genesee Scientific, San Diego, CA, United States) was used to visualize the antibody–antigen interactions, and membranes were digitally imaged using the iBright 1500 system (Invitrogen, Carlsbad, CA, United States). Immunoblots were analyzed by measuring each band’s integrated optical density (IOD) with ImageJ software (National Institutes of Health, Bethesda, MD, United States).

### RNA Isolation, cDNA Synthesis, and Real-Time PCR

Total RNA was isolated from C2C12 myotubes using TRIzol reagent (Invitrogen, Carlsbad, CA, United States) per manufacturer’s guidelines and as previously described (White et al., 2009). After phenol–chloroform extraction, RNA was purified using the PureLink^®^ RNA Mini Kit (Invitrogen, Carlsbad, CA, United States) and eluted in RNAase-free water. Total RNA concentration (260 nm) and purity (260/280 ratio) were measured using UV spectrophotometry, and total RNA was stored at −80°C. cDNA was reverse transcribed from 1 μg of total RNA using Superscript IV Reverse Transcriptase and random hexamers according to manufacturer guidelines (#18090200; Invitrogen) in a final volume of 20 μl at 23°C for 10 min, 50°C for 10 min, and 80°C for 10 min. cDNA was stored at −80°C. Real-time PCR was performed using reagents from Applied Biosystems (Foster City, CA, United States). Gene expression was carried out in 20-μl reactions using 2 × PowerTrack SYBR Green master mix, 2 μl of cDNA, 1 μl of forward and reverse primers (500 nM), and nuclease-free H_2_O. The sequences of the primers were verified using NIH Primer Blast software, as follows: MyHC-Slow Type I (Myh7) [NM_080728.3]: Fw 5′-CCAAGGGCCTGAATGAGGAG-3′, Rv 5′-GCAAAGGCTCCAGGTCTGAG-3′; MyHC-Fast type IIA (Myh2) [NM_001039545.2]: Fw 5′-AGGCGGCTGAGGAGC ACGTA-3′, Rv 5′-GCGGCACAAGCAGCGTTGG-3′; MyHC- Fast type IIB (Myh4) [NM_010855.3]: Fw 5′-CACCTGG ACGATGCTCTCAGA-3′, Rv 5′-GCTCTTGCTCGGCCACTCC -3′; MyHC-Fast type IIX (Myh1) [NM_030679.2]: Fw 5′-GAGG GACAGTTCATCGATAGCAA-3′, Rv 5′-GGGCCAACTTGTCA TCTCTCAT-3′; skeletal muscle alpha actin (Acta1) [NM_00127 2041.1]: Fw 5′-CTCCTACGTGGGTGATGAGG-3′, Rv 5′-AGGTGTGGTGCCAGATCTTC-3′; myogenin (Myog) [NM _031189.2]: Fw 5′-GCACTGGAGTTCGGTCCCAA-3′, Rv 5′-TA TCCTCCACCGTGATGCTG-3′; MyoD (Myod1) [NM_010866. 2]: Fw 5′-GAGATGCGCTCCACTATGCT-3′, Rv 5′-TGGCAT GATGGATTACAGCG-3′; Myocyte Enhancer Factor 2C (MEF 2C) [NM_001170537.1]: Fw 5′-GAGCCGGACAAACTCAGA CA-3′ Rv 5′-GGCTGTGACCTACTGAATCGT-3′; and GAPDH [NM_001289726]: Fw 5′-ACCACAGTCCATGCCATCAC-3′ Rv 5′-TCCACCACCCTGTTGCTGTA-3′. Primer sequences were synthesized by Integrated DNA Technologies (IDT, Coralville, IA, United States) and validated via agarose gel electrophoresis. RT-PCR was carried out on the Applied Biosystems QuantStudio3 system. Reactions were incubated for 2 min at 50°C, 10 min at 95°C, followed by 40 cycles of a 15-s denaturation step at 95°C, and 1-min annealing/extension at 60°C. The 2^–ΔΔ*Ct*^ method (Livak and Schmittgen, 2001) was used to determine gene expression changes between treatment groups with the GAPDH Ct as the correction factor.

### Statistical Analysis

All experiments included a minimum of three replicates from at least two independent experiments. Results are expressed as mean ± SEM. Student’s unpaired *t*-test, one-way ANOVA, and two-way ANOVA (indicated in the figure legends) were used to examine the effects of stretch and culture conditions. Tukey’s *post hoc* multiple comparisons test was used when a significant interaction was present. *p*-Values ≤0.05 were considered significant.

## Results

### Effects of Colon-26 Tumor Cell Conditioned Media in C2C12 Myotubes Grown on Rigid Polystyrene Substratum

**FIGURE 1:**
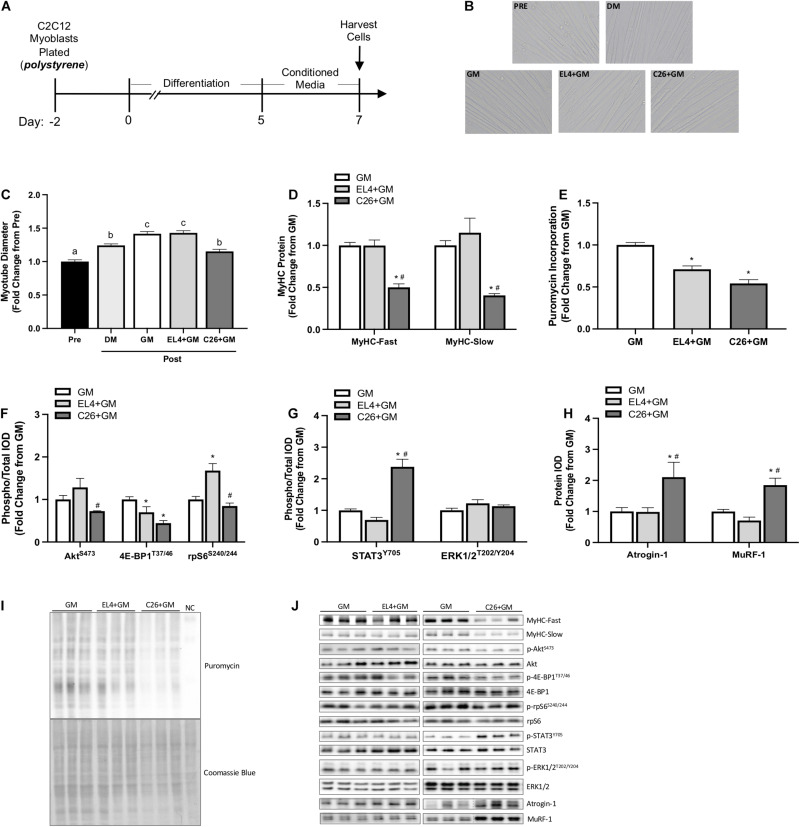
Effects of tumor cell conditioned media on C2C12 myotube size and protein turnover signaling on type I collagen-coated polystyrene plates. **(A)** Experiment overview: C2C12 myoblasts are plated on type I collagen-coated rigid polystyrene plates in growth media (GM); after 48 h at ∼85% confluence, differentiation media (DM) is added, which corresponds to day 0 of differentiation. At 5 days post-differentiation, myotubes are imaged (Pre) and then 50% tumor cell conditioned media (CM) is diluted with serum-free DMEM and added to wells for 48 h. Conditioned media is collected from cachectic colon-26 (C26 + GM), non-cachectic EL4 lymphoma (EL4 + GM), or growth media with no cells, which is used as a media control (GM). Myotubes maintained in DM for 48 h are used as a control for myotube growth. Cells are harvested at day 7 post-differentiation for protein. **(B)** Representative × 20 brightfield myotube images. **(C)** Myotube diameter Pre (day 5; *n* = 216 myotubes) and Post (day 7; *n* = 162–216 myotubes/group) 48 h DM, GM, or CM incubation. **(D)** Myosin heavy chain (MyHC)-Fast (type II fibers) and MyHC-Slow (type I fibers) protein expression measured by Western blotting. **(E)** Quantified protein synthesis rate measured by Western blotting against puromycin (*n* = 6–12/group). Western blotting analyses of panel **(F)** ratio of phosphorylated (p-) to total protein expression of Akt^*S*473^ (*n* = 6/group), 4E-BP1^*T*37/46^ (*n* = 6/group), and rpS6^*S*240/244^ (*n* = 6/group). **(G)** Ratio of phosphorylated (p-) to total protein expression of STAT3^*Y*705^ (*n* = 6–12/group) and ERK1/2^*T*202/204^ (*n* = 6/group) and **(H)** Atrogin-1/MAFbx and MuRF-1 (*n* = 6/group). **(I)** Representative Western blotting image displaying puromycin incorporation and corresponding Coomassie blue stain; NC is the negative control protein sample from C2C12 myotubes without puromycin treatment. **(J)** Representative Western blotting images, separate panels are from separate blots each ran with GM control, and dashed lines represent different areas of the same gel. Data are presented as means ± SEM as a fold change from GM. One-way ANOVA was performed to determine differences. Lowercase letters (a, b, and c) denote significant difference between groups; *significant from GM control; ^#^significant from EL4 + GM. Statistical significance set at *p* ≤ 0.05.

We examined the effects of tumor cell CM under standard culture conditions. GM, C26 CM (C26 + GM), or EL4 CM (EL4 + GM) was administered to 5-day differentiated myotubes grown on type I collagen-coated polystyrene plates for 48 h (Figure 1A). EL4 lymphoma cells that do not induce cachexia (Zhang et al., 2017) were used as a tumor cell CM control. Myotubes were imaged for diameter Pre (day 5) and Post (day 7) CM exposure (Figure 1B). Myotubes maintained in DM were imaged and used as a control for myotube growth. Under standard DM culture conditions, myotubes increased diameter from day 5 to day 7 (24%; *p* < 0.0001) (Figure 1C). EL4 + GM-treated myotubes significantly increased diameter compared to Pre (*p* < 0.0001), and this increase was equivalent to GM treatment (42%) (Figure 1C). C26 + GM-treated myotubes significantly increased diameter compared to Pre (15%). However, they were significantly smaller in size than both GM and EL4 + GM-treated myotubes (Figure 1C). Along with decreased myotube size, C26 + GM-treated myotubes had significantly lower protein expression of both MyHC-Fast (*p* < 0.001, −50%) and MyHC-Slow (*p* = 0.007, −60%) isoforms compared with the GM and EL4 + GM conditions (Figure 1D). These results demonstrate that C26 tumor-derived factors can suppress myotube growth and MyHC protein expression and that these effects are specific to cachectic tumor-derived factors.

Altered muscle protein turnover regulation contributes to cachexia development (Baracos, 2000), and tumor-derived factors can disrupt this regulation in cultured myotubes (Gao and Carson, 2016; Gao et al., 2017; Chiappalupi et al., 2020). Both EL4 and C26 tumor-derived factors displayed significant reductions in protein synthesis rate measured by puromycin incorporation (EL4 + GM: −29%, C26 + GM: −46%) (Figures 1E,I). Compared with EL4 + GM, C26 + GM-treated myotubes displayed a significant reduction in the phosphorylation of Akt (S473) (*p* = 0.025) and rpS6 (S240/244) (*p* < 0.001); both EL4 + GM (*p* = 0.047) and C26 + GM (*p* < 0.001) tumor-derived factors significantly reduced the phosphorylation of 4E-BP1 (T37/46) (Figures 1F,J). C26 + GM induced STAT3 phosphorylation (Y705) 138% compared with controls (*p* < 0.001), and ERK1/2 phosphorylation was unchanged across all conditions (Figures 1G,J). Under standard culture conditions, 48 h incubation with C26 + GM significantly induced Atrogin-1 (*p* = 0.009, 111%) and MuRF-1 (*p* < 0.001, 85%) as compared with GM and EL4 + GM (Figures 1H,J). These data suggest that C26 tumor-derived factors can alter protein turnover signaling involving Akt/mTORC1 signaling (i.e., 4E-BP1 and rpS6) and ubiquitin–proteasome-mediated breakdown.

### Myotube Growth Suppression by Colon-26 Tumor-Derived Factors on Flexible Silastic Membranes

**FIGURE 2:**
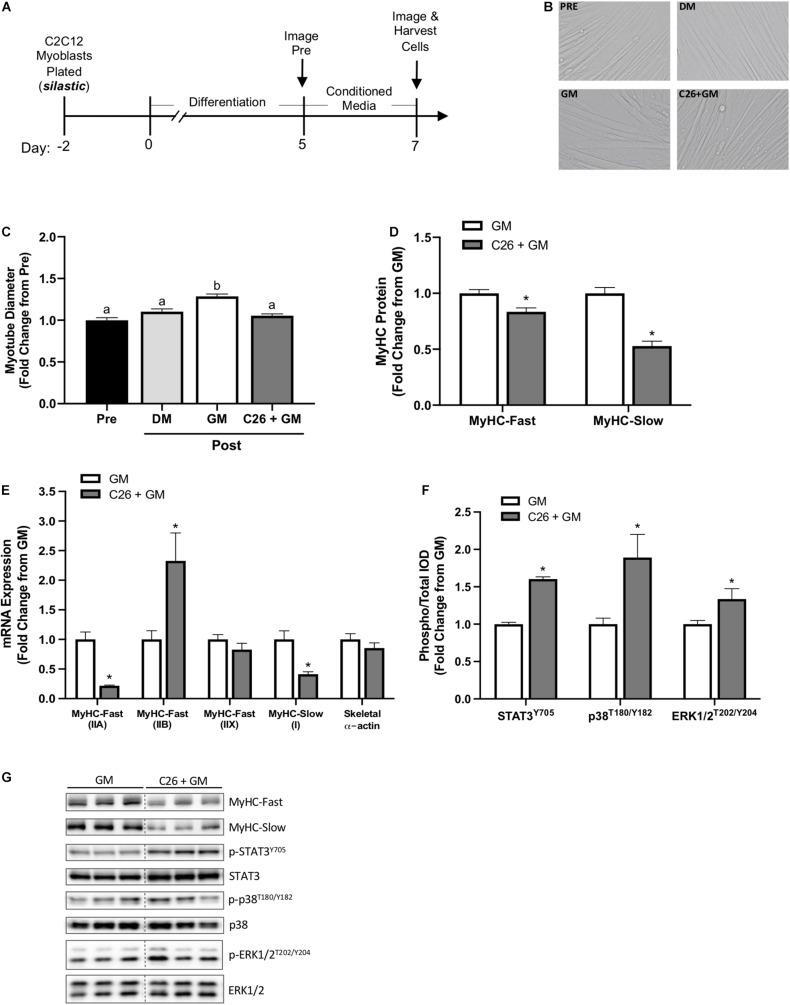
C2C12 myotube growth suppression by colon-26 (C26) tumor-derived factors on type I collagen-coated silastic membranes. **(A)** Experiment overview: C2C12 myoblasts are plated on type I collagen-coated silastic Uniflex membranes in growth media (GM). After 48 h at ∼85% confluence, differentiation media (DM) is added, which corresponds to day 0 of differentiation. On day 5 of differentiation, myotubes are fully formed, and conditioned media (CM: GM, C26 + GM) diluted with 50% serum-free DMEM is added to wells for 48 h. Myotubes are imaged Pre and Post 48-h incubation with DM, GM, or C26 + GM for diameter measurements. Cells are harvested for protein and RNA on day 7 of differentiation. **(B)** Representative × 20 brightfield images. **(C)** Myotube diameter quantification Pre (day 5; *n* = 162 myotubes) and Post 48 h DM, GM, or C26 + GM incubation (*n* = 162–378 myotubes/group). **(D)** Myosin heavy chain (MyHC)-Fast (type II fibers) and MyHC-Slow (type I fibers) protein expression measured by Western blotting (Fast: *n* = 18–21/group; Slow: *n* = 9/group). **(E)** MyHC-Fast type IIA (Myh2), type IIB (Myh4), type IIX (Myh1), MyHC-Slow type I (Myh7), and skeletal muscle α-actin (Acta1) mRNA expression (*n* = 6/group) calculated using the 2^–ΔΔ*Ct*^ method with GAPDH as a housekeeping gene. **(F)** Ratio of phosphorylated (p-) to total protein expression of signal transducer and activator of transcription 3 (STAT3^*Y*705^) (*n* = 12–15/group); p38^*T*180/Y182^ mitogen-activated protein kinase (MAPK) (*n* = 6/group), and extracellular signal-regulated kinase 1/2 (ERK1/2^*T*202/Y204^) (*n* = 6/group) measured by Western blotting. **(G)** Representative Western blotting images: dashed line represents different areas of the same gel. Data are presented as means ± SEM as a fold change from GM. One-way ANOVA **(C)** or Student’s unpaired *t*-test was performed to determine differences. Lowercase letters (a, b, and c) denote significant difference between groups; *significant from GM control. Statistical significance set at *p* ≤ 0.05.

Growth media or C26 tumor cell CM (C26 + GM) was administered to myotubes at day 5 of differentiation for 48 h on type I collagen-coated silastic membranes (Figure 2A). Myotubes were imaged for diameter at day 5 (Pre) and day 7 (Post) (Figure 2B). Switching C2C12 myotubes from DM (2% horse serum) to GM (5% FBS) has been shown to induce myotube growth (von Walden et al., 2016). GM significantly increased myotube diameter compared with DM (*p* < 0.001) (Figure 2C). Compared with Pre, GM treatment increased myotube diameter 29% (*p* < 0.001), and C26 + GM-treated myotubes did not change (*p* = 0.618) myotube diameter (Figure 2C). These results demonstrate that C26 tumor-derived factors can suppress myotube growth measured by diameter despite a high growth factor environment.

Myosin heavy chain isoform protein expression was quantified on day 7 after 48 h of GM or C26 + GM exposure. Increased MyHC isoform expression is critical for myotube differentiation and growth (Brown et al., 2012). We report that C26 CM can decrease myotube MyHC protein concentration in myotubes grown on flexible silastic membranes. C26 + GM-treated myotubes decreased MyHC-Fast protein expression −17% (*p* = 0.002) and MyHC-Slow protein expression −47% (*p* < 0.0001) (Figure 2D). We next examined if C26 tumor-derived factors regulate changes in myotube MyHC expression at the mRNA level. mRNA expression was quantified at day 7 after 48 h of GM or C26 + GM exposure. C26 CM induced isoform-specific shifts in MyHC mRNA expression (Figure 2E). The expression of MyHC-Fast type IIA (Myh2) mRNA was reduced by −78% (*p* < 0.0001), type IIB (Myh4) mRNA was increased by 133%, type IIX (Myh1) did not significantly change, and MyHC-Slow type I (Myh7) was reduced by -59% (*p* = 0.003) by the C26 + GM treatment (Figure 2E). Skeletal α-actin is a principle component of muscle thin filaments and interacts with the MyHC proteins for contraction (Sparrow et al., 2003). Forty-eight hours of C26 + GM incubation did not alter skeletal α-actin mRNA expression (Figure 2E). Therefore, C26-derived factors reduce the concentration of both MyHC protein and mRNA in myotubes, and this preferential loss of MyHC has potential implications for both size and contractile function.

C26 CM can activate multiple signaling pathways associated with myotube catabolic signaling (Seto et al., 2015). We examined several proteins that can regulate muscle growth and are implicated in cancer cachexia, including STAT3 (Puppa et al., 2014), p38 MAPK (Ding et al., 2017; Brown et al., 2018), and ERK1/2 (Gao and Carson, 2016; VanderVeen et al., 2018). Despite incubation in a high serum growth-promoting environment, C26 + GM induced a 57% increase in the phosphorylation of STAT3 (Y705) (*p* < 0.001), an 89% increase (*p* = 0.011) in p38 (T180/Y182) phosphorylation, and a 34% increase (*p* = 0.033) in ERK1/2 (T202/Y204) phosphorylation (Figures 2F,G).

### Mechanical Stretch Effects on Growth and Myosin Protein Expression in Colon-26 Treated Myotubes

**FIGURE 3:**
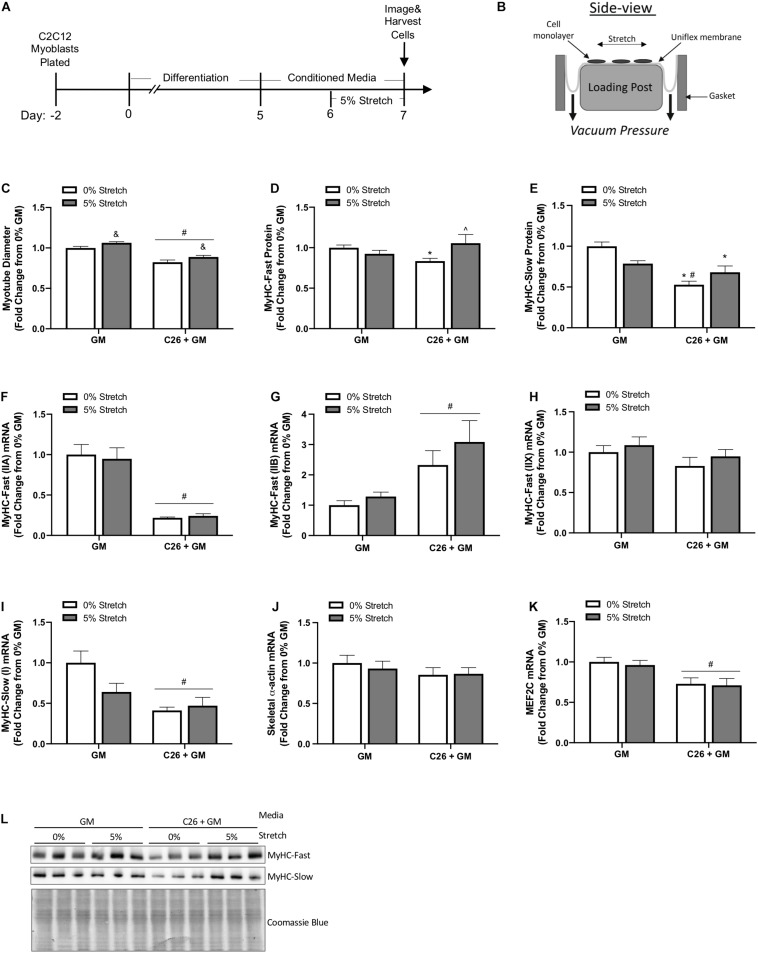
Effects of mechanical stretch on growth and myosin expression in colon-26 (C26)-treated myotubes. **(A)** Experiment overview: C2C12 myoblasts are plated on in growth media (GM); after 48 h at ∼85% confluence, differentiation media is added, which corresponds to day 0. At day 5 of differentiation, fully formed contractile myotubes are present, and conditioned media (CM; GM or C26 + GM) is diluted with 50% serum-free DMEM and added to wells for 48 h. Myotubes are either left unstretched (0%) or stretched 5% in the last 24 h of CM incubation. **(B)** The side view of the stretching device; cells adhere to the type I collagen coated Uniflex membrane, and a rubber gasket is placed around each six-well plate to create a tight seal; vacuum pressure is applied to deform the membrane around the loading post, which creates a uniaxial stretch (image adapted from Flexcell International). **(C)** Myotube diameter of unstretched (0%) and stretched (5%) myotubes treated with GM control or C26 + GM (*n* = 216 myotubes/group). **(D)** Myosin heavy chain (MyHC)-Fast (0% *n* = 18–21/group; 5% *n* = 9/group) and **(E)** MyHC-Slow (*n* = 9–11/group) protein expression measured by Western blotting. **(F)** MyHC-Fast (type IIA, Myh2) (*n* = 6/group), **(G)** MyHC-Fast (type IIB, Myh4) (*n* = 6/group), **(H)** MyHC-Fast (type IIX, Myh1) (*n* = 6/group), **(I)** MyHC-Slow (type I, Myh7) (*n* = 6/group), **(J)** skeletal α-actin (Acta1) (*n* = 6/group), and **(K)** Myocyte Enhancer Factor 2C (MEF2C) (*n* = 6/group) mRNA expression calculated using 2^–ΔΔ*Ct*^ method with GAPDH as a housekeeping gene. **(L)** Representative Western blotting images with Coomassie blue stain as a protein loading control. Data are presented as means ± SEM as a fold change from GM 0% stretch. Two-way ANOVAs were performed to determine differences. ^&^Main effect of stretch; ^#^main effect of C26 + GM; *significant from GM 0% stretch; ^#^significant from GM 5% stretch; ^significant from C26 + GM 0% stretch. Statistical significance set at *p* ≤ 0.05.

Mechanical signaling induced by chronic passive stretch is a potent inducer of myotube growth and anabolic signaling (Vandenburgh and Kaufman, 1979; Gao and Carson, 2016). Additionally, dynamic stretch occurring with the administration of C26-derived catabolic factors can promote increased myotube diameter (Baccam et al., 2019). Our study extends this understanding by examining chronic stretch application after the initiation of catabolism inducing C26 CM. We assessed if stretch could induce a growth stimulus in myotubes already exposed to C26 cachectic factors. Furthermore, we examined the effects of stretch in a high serum growth-promoting state. Myotubes were uniaxially stretched 5% after 24 h of exposure to C26 + GM and then maintained in the stretched state in C26 + GM for 24 h (Figure 3A). There was a main effect for myotubes treated with C26 + GM for 48 h to decrease (*p* < 0.0001) myotube diameter (Figure 3C). However, there was a main effect for the 24-h stretch treatment to increase (*p* = 0.003) myotube diameter in both GM and C26 + GM conditions (Figure 3C). These results provide evidence that stretch can induce a growth stimulus in myotubes that are in a catabolic state and in a growth factor-rich environment (5% FBS) that generated growth in the absence of tumor-derived factors.

We next examined if 24 h of stretch could rescue suppressed MyHC-Fast and MyHC-Slow protein expression in myotubes incubated with C26 CM. We report that stretch has different effects on fast and slow MyHC protein expression. Stretch increased MyHC-Fast protein expression (26%, *p* = 0.023) in C26 + GM myotubes compared with unstretched C26 + GM myotubes (Figures 3D,L). Furthermore, MyHC-Fast protein expression in stretched C26 + GM myotubes was not different than GM levels, indicating that stretch had rescued MyHC-Fast protein expression. Interestingly, stretch did not change (*p* = 0.251) MyHC-Slow protein expression in C26 + GM incubated myotubes, and unstretched (0%) C26 + GM-treated myotubes had significantly lower MyHC-Slow protein expression than both unstretched (*p* < 0.001) and stretched (*p* = 0.021) GM-treated myotubes (Figures 3E,L). We also examined the effect of stretch on MyHC mRNA expression. We measured MyHC-Fast type IIA, IIB, IIX, and MyHC-Slow type I mRNA expression after 24 h of stretch. Stretch did not change the C26 + GM suppression MyHC-Fast type IIA (Figure 3F) or MyHC-Slow type I (Figure 3I) mRNA expression. Likewise, in both GM and C26 conditions, passive stretch did not alter mRNA expression of MyHC-Fast type IIB (Figure 3G), type IIX (Figure 3H), and skeletal muscle α-actin (Figure 3J). There were main effects for C26 + GM to decrease MyHC-Fast type IIA (*p* < 0.001) (Figure 3F), increase type IIB (*p* = 0.002) (Figure 3G), and decrease MyHC-Slow type I (*p* = 0.002) (Figure 3I) mRNA expression. MEF2C is an important transcriptional regulator of MyHC genes and can regulate thick filament assembly in myotubes (Hinits and Hughes, 2007; Piasecka et al., 2021). There was a main effect for C26 + GM to decrease (27%; *p* = 0.001) MEF2C mRNA expression (Figure 3K). Stretch did not change MEF2C mRNA expression (*p* = 0.687). These data suggest that stretch-induced mechanical signaling can rescue suppressed MyHC-Fast protein expression in myotubes undergoing catabolism initiated by C26 tumor-derived factors. Interestingly, this effect was independent of changes in MyHC mRNA expression and specific to MyHC-Fast protein. Suppressed MyHC-Slow protein expression by C26 CM was not responsive to stretch-induced mechanical signaling.

### Chronic Mechanical Stretch Effects on Protein Turnover Signaling in Colon-26 Treated Myotubes

**FIGURE 4:**
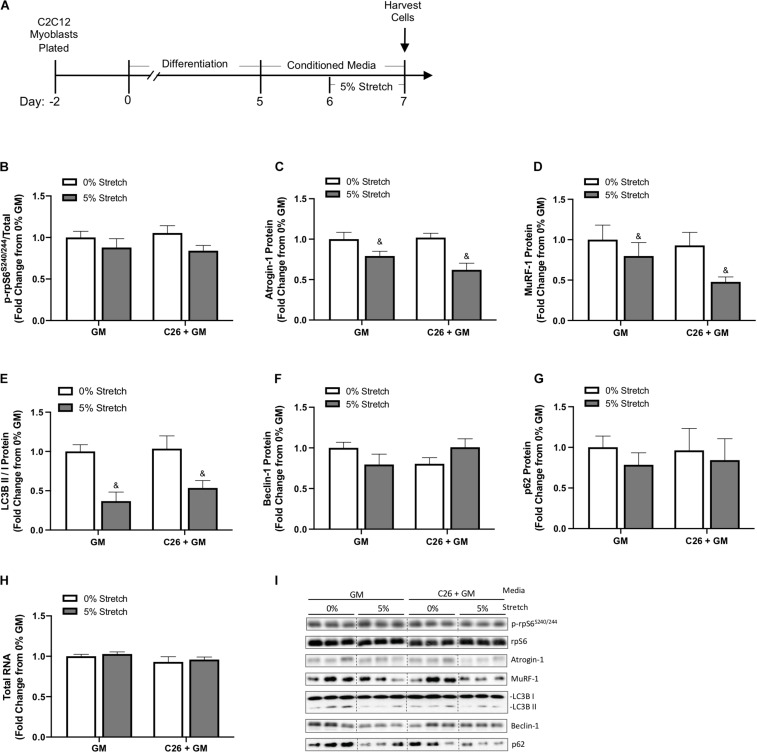
Effects of 24-h stretch on growth related signaling in myotubes treated with colon-26 (C26) tumor-derived factors. **(A)** Experiment overview: C2C12 myoblasts are plated on Uniflex membranes in growth media (GM); after 48 h at ∼85% confluence, differentiation media is added, which corresponds to day 0. Myotubes are differentiated for 5 days upon which 50% conditioned media (CM; GM or C26 + GM) is diluted with serum-free DMEM and added to wells for 48 h; myotubes are either left unstretched (0%) or stretched 5% in the last 24 h of CM incubation. Western blotting analyses of **(B)** ratio of phosphorylated (p-) to total protein expression of ribosomal protein S6 (rpS6^*S*240/244^) (*n* = 6/group), **(C)** Atrogin-1/muscle atrophy F-box (MAFbx) (*n* = 6/group), **(D)** Muscle RING finger 1 (MuRF-1) (*n* = 6/group), **(E)** microtubule-associated protein light chain 3B (LC3B) II/I ratio (*n* = 6–12/group), **(F)** Beclin-1 (*n* = 6/group), and **(G)** p62/SQSTM1 (*n* = 6/group) protein expression. **(H)** Total RNA content (*n* = 6/group). **(I)** Representative Western blotting images; dashed line represents different areas of the same gel. Data are presented as means ± SEM as a fold change from GM 0% stretch. Two-way ANOVAs were performed to determine differences. ^&^Main effect of stretch. Statistical significance set at *p* ≤ 0.05.

Skeletal muscle atrophy is associated with suppressed mTORC1 signaling, disrupted autophagy flux, and decreased RNA content, diminishing ribosomal capacity for protein synthesis (Bhogal et al., 2006). E3 ligases Atrogin-1/MAFbx and MuRF-1 are involved in ubiquitin–proteasome degradation and have an established role in muscle atrophy (Lecker et al., 1999; Bodine et al., 2001; Kandarian and Stevenson, 2002). Therefore, we assessed these factors to gain insight into the stretch regulation of myotube growth and protein turnover after exposure to C26 tumor-derived factors (Figure 4A). We first measured the phosphorylation of ribosomal protein S6 (rpS6), one downstream marker of the mammalian target of rapamycin (mTOR) pathway (Mieulet et al., 2007). rpS6 phosphorylation (S240/244) was unchanged by C26 + GM and stretch (Figures 4B,I). Skeletal muscle-specific E3 ubiquitin ligases Atrogin-1/MAFbx and MuRF-1 can degrade myosin (Clarke et al., 2007; Cohen et al., 2009; Bodine and Baehr, 2014). Interestingly, we observed a main effect for 24 h of stretch to decrease Atrogin-1 and MuRF-1 protein expression in both GM and C26 + GM-treated myotubes (Figures 4C,D,I). We next examined established markers of autophagy flux, including microtubule-associated protein light chain 3 (LC3B) I and II, Beclin-1, and p62/SQSTM1 (Mizushima and Yoshimori, 2007; Penna et al., 2013). We observed a main effect for chronic stretch to decrease the LC3B II/I ratio (*p* < 0.001) (Figures 4E,I) with no changes in Beclin-1 or p62 protein expression (Figures 4F,G,I). Total RNA content in myotubes remained unchanged across all conditions (Figure 4H). These data suggest that chronic stretch may protect myosin content by decreasing the expression of Atrogin-1 and MuRF-1 and reduced autophagy flux, rather than the induction of anabolic signaling involving total RNA and mTOR signaling relating to rpS6.

### Stretch Effect on Mechanical and Growth-Related Factors After Colon-26 Conditioned Media Exposure

**FIGURE 5:**
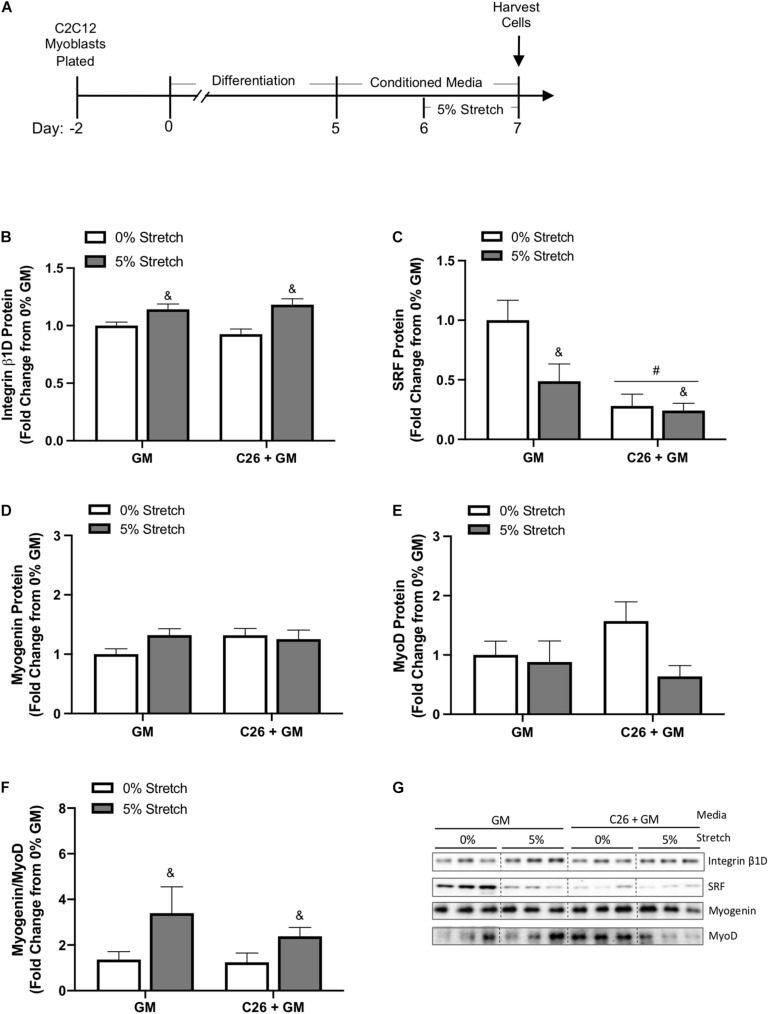
Effects of 24-h stretch mechanical and growth-related signaling in colon-26 (C26)-treated myotubes. **(A)** Experiment overview: C2C12 myoblasts are plated on Uniflex membranes in growth media (GM); after 48 h at ∼85% confluence, differentiation media is added corresponding to day 0. Myotubes are differentiated for 5 days upon which 50% conditioned media (CM; GM or C26 + GM) is diluted with serum-free DMEM and added to wells for 48 h; myotubes are either left unstretched (0%) or stretched 5% in the last 24 h of CM incubation. Immediately after stretch, cells are harvested for protein. Western blotting analyses of **(B)** integrin β1D (*n* = 9/group), **(C)** serum response factor (SRF) (*n* = 9/group), **(D)** myogenin (*n* = 6/group), and **(E)** MyoD (*n* = 6/group) protein expression. **(F)** Ratio individual protein expression of myogenin, a marker of terminal myoblast differentiation, to MyoD, a marker of myoblast proliferation and initiation of differentiation (*n* = 6/group). **(G)** Representative Western blotting images; dashed line represents different areas of the same gel. Data are presented as means ± SEM as a fold change from GM 0% stretch. Two-way ANOVAs were performed to determine differences. ^&^Main effect of stretch, ^#^main effect of C26 + GM. Statistical significance set at *p* ≤ 0.05.

We investigated the effects of 24 h stretch on various regulators of muscle growth during C26 CM incubation (Figure 5A). We observed a main effect for mechanical stretch to increase integrin β1D protein expression (*p* < 0.001) (Figure 5B). SRF is a member of the MADS-box family of transcription factors important for growth and myogenic processes (Li et al., 2005). Moreover, mechanical stretch can induce SRF mRNA expression (Carson and Booth, 1999). Surprisingly, there was a main effect for both stretch (*p* = 0.035) and C26 tumor-derived factors (*p* < 0.001) to decrease SRF protein expression (Figures 5C,G). We further examined the role of stretch and C26 + GM on myogenesis by measuring the ratio of myogenin, which is expressed in late stages of differentiation, to MyoD, a marker of myoblast proliferation and initiation of differentiation. The relative mRNA expression of myogenin (GM 0%, 1.00 ± 0.03; GM 5%, 1.03 ± 0.09; C26 + GM 0%, 0.809 ± 0.08; and C26 + GM 5%, 1.01 ± 0.08) and MyoD (GM 0%, 1.02 ± 0.10; GM 5%, 0.905 ± 0.12; C26 + GM 0%, 0.752 ± 0.07; and C26 + GM 5%, 0.842 ± 0.10) was unchanged. Due to variability in protein expression, myogenin (Figure 5D) and MyoD (Figure 5E) individual protein expression was similar across groups. To account for variability between samples, we calculated the ratio of myogenin-to-MyoD protein expression in each sample. There was a main effect for stretch to increase the myogenin-to-MyoD ratio (*p* = 0.027) (Figure 5F). These results provide evidence that regardless of C26 tumor-derived factor incubation, stretch can increase integrin β1D protein expression to modulate intrinsic mechanical signaling. These changes are independent of stretch-induced increases in SRF protein expression and associated with an effect for stretch to increase the myogenin-to-MyoD protein ratio.

### Acute Stretch Effects on Mechanical and Colon-26-Induced C2C12 Myotube Signaling

**FIGURE 6:**
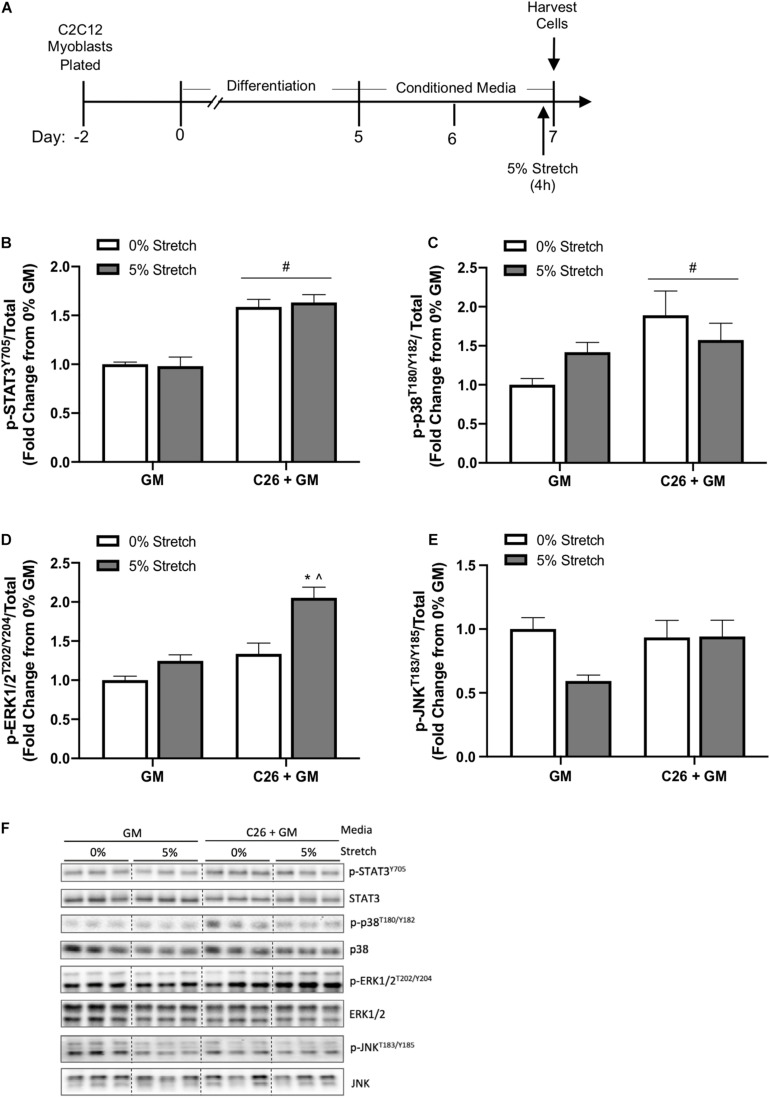
Effects of 4-h stretch on colon-26 (C26) conditioned media-induced myotube signaling pathways. **(A)** Experiment overview: C2C12 myoblasts are plated on in growth media (GM); after 48 h at ∼85% confluence, differentiation media is added, which corresponds to day 0. At day 5 of differentiation, conditioned media (CM; GM or C26 + GM) is diluted with 50% serum-free DMEM and added to wells for 48 h. Myotubes are either left unstretched (0%) or stretched 5% in the last 4 h of CM incubation. Immediately after stretch, cells are harvested for protein. Western blotting analyses of the ratio of phosphorylated (p-) to total protein expression of **(B)** signal transducer and activator of transcription 3 (STAT3^*Y*705^) (*n* = 9–12/group), **(C)** p38^*T*180/Y182^ MAPK (*n* = 6/group), **(D)** extracellular signal-regulated kinase 1/2 (ERK1/2^*T*202/Y204^) (*n* = 6/group), and **(E)** c-Jun N-terminal kinase (JNK^*T*183/Y185^) (*n* = 9/group) protein expression. **(F)** Representative Western blotting images; dashed line represents different areas of the same gel. Data are presented as means ± SEM as a fold change from GM 0% stretch. Two-way ANOVAs were performed to determine differences. ^#^Main effect of C26 + GM; *significant from GM 0% stretch, ^significant from C26 + GM 0% stretch. Statistical significance set at *p* ≤ 0.05.

In a separate experiment, we examined the effects of acute 4-h stretch on cachectic and growth-related signaling in C26 + GM-treated myotubes. Several cytokines associated with cancer-induced wasting can stimulate muscle STAT3 phosphorylation (Bonetto et al., 2011; Puppa et al., 2014; Zimmers et al., 2016). Cancer cachexia can induce muscle p38 MAPK, ERK1/2, and JNK signaling (Brown et al., 2018; Mulder et al., 2020). Interestingly, mechanical signaling can also activate these pathways (Kumar et al., 2002; Martineau and Gardiner, 2002; Gao and Carson, 2016). Several well-characterized stretch-activated signaling pathways increase at the onset of chronic stretch and then return to baseline (Zhan et al., 2007). Therefore, we examined 4 h of stretch to determine acute activation of these signaling pathways. Myotubes were stretched 5% for the last 4 h of either the GM or C26 + GM treatment (Figure 6A). There was no effect for stretch to change STAT3 (Y705) phosphorylation (*p* = 0.865) (Figure 6B), p38 (T180/Y182) phosphorylation (*p* = 0.809) (Figure 6C), or JNK (T183/Y185) phosphorylation (*p* = 0.067) (Figures 6E,F). A preplanned analysis determined the effect of stretch in the GM condition; stretch induced a 42% increase in p38 (T180/Y182) (*p* = 0.014) phosphorylation and a 25% increase in ERK1/2 (T202/Y204) (*p* = 0.015) phosphorylation. Interestingly, this signaling activation occurred within the high serum environment. Stretch-induced ERK1/2 phosphorylation was further induced 54% in C26 + GM-treated stretched cells (*p* < 0.001) (Figures 6D,F). These data suggest that C26 + GM does not blunt the stretch response of p-p38 and p-ERK1/2 and that acute static stretch had no effect on the C26 CM induction of STAT3 phosphorylation, which was similarly elevated with 4 h of stretch.

## Discussion

Colon-26 carcinoma represents a well-characterized and extensively used preclinical cancer cachexia model (Bonetto et al., 2016). *In vitro*, C26 CM produces myotube atrophy, and tumor-derived factors in the CM can disrupt the regulation of myotube protein turnover (Jackman et al., 2017; Kandarian et al., 2018; Liu et al., 2019; Chiappalupi et al., 2020; Schmidt et al., 2020; Wang et al., 2021). Dynamic exercise can prevent metabolic and functional disruptions and suppress signaling associated with cachexia (Halle et al., 2020). Mechanical stimulation of muscle via passive stretch can promote muscle growth and functional adaptations (Carson et al., 1995; De Deyne, 2001; Zöllner et al., 2012). Skeletal muscle MyHC is a critical protein involved in skeletal muscle force generation and function (Weiss et al., 1999). Cancer can cause MyHC loss in muscle and myotubes (Acharyya et al., 2004; Guigni et al., 2018; Liu et al., 2019; Yamada et al., 2020). While our mechanistic understanding of cancer cachexia has advanced significantly, critical gaps remain in our knowledge of constraints that cachectic factors place on intrinsic muscle properties such as mechanotransduction. How stretch-induced mechanical signaling impacts the regulation of MyHC expression after exposure to cachectic tumor-derived factors is uncertain. Therefore, we sought to examine whether mechanical signaling induced by both chronic and acute passive stretch can impact C2C12 myotube growth and MyHC expression after incubation with C26 CM. Our data provide intriguing evidence that myotubes pretreated with C26 CM can still respond to mechanical stimuli associated with passive stretch. The induction of mechanical signaling provides a growth stimulus and maintains MyHC-Fast protein expression, independent of changes in MyHC mRNA expression changes. These benefits correspond to increased integrinβ1D expression, a stretch activation of ERK1/2, increased myogenin/MyoD ratio, and the suppression of Atrogin-1/MAFbx, MuRF-1, and LC3B II/I ratio.

*In vitro* studies have consistently reported that tumor-derived factors can reduce myotube size. Higher tumor-CM concentrations can decrease diameter in a dose-dependent manner (Pin et al., 2018; Zhong et al., 2019). As in the present study, myotube atrophy mechanisms have been investigated with 50% CM. However, this dilution of tumor-derived media can provide a serum-rich growth environment of myotubes consisting of 5% FBS. Tumor cells, such as C26 and EL4 cells, are cultured in serum-rich media (10% FBS). Therefore, increasing the percentage of CM also increases the serum concentration and modifies C2C12 myotube culture conditions compared with standard differentiation conditions (e.g., 2% horse serum). Mature myotubes exposed to high serum elicit a robust growth response (von Walden et al., 2016), which aligns with our results of significant growth of myotubes treated with 50% GM. These results held under two different culture conditions, including myotubes plated on rigid polystyrene collagen coated plates and flexible silastic membranes coated with collagen. Interestingly, despite a high growth factor environment, myotubes exposed to C26 CM exhibit suppressed growth, which was not observed with EL4 CM, suggesting that this response is specific to factors implicated in cachexia. Inflammatory factors such as interleukin-6 (IL-6), tumor necrosis factor (TNF)-α, and leukemia inhibitory factor (LIF) are commonly examined for their roles in cancer-induced muscle wasting (Carson and Baltgalvis, 2010; Patel and Patel, 2017). It has been previously described that C26 media contains low levels of IL-6 and TNF-α and high levels of LIF, which are thought to be a key factor for C26 atrophy *in vivo* and *in vitro* (Seto et al., 2015; Kandarian et al., 2018). In line with previously published data, C26 secreted factors induced STAT3 phosphorylation under both plating conditions, indicating an important role for STAT signaling in C26 cachexia. CM experiments are regularly utilized to examine the effects of tumor-derived factors on skeletal muscle myotubes. However, it is important to note that this *in vitro* model is a biological assay and does not precisely mimic preclinical and patient serum concentrations of secreted factors. Mechanical stimulation of myotubes via dynamic and static stretch can increase myotube diameter even in the presence of tumor-derived factors (Gao and Carson, 2016; Baccam et al., 2019). While myotubes incubated with C26 CM suppressed growth over the 48 h period, stretch in the last 24 h promoted growth, although to a lesser extent than 5% FBS GM. Further research is warranted to examine this differential growth response to anabolic stimuli in myotubes treated with tumor-derived factors and the potential effects that high serum with tumor-derived factors may have on muscle signaling.

Cachectic tumor-derived factors can produce atrophy by altering protein turnover signaling. We observed suppression of protein synthesis, Akt/mTORC1 signaling, and induction of E3 ubiquitin ligases under standard culture conditions in response to tumor-derived factors. C26 CM induces the phosphorylation of p38 MAPK and ERK1/2, which are well-characterized signaling molecules implicated in cancer cachexia (Seto et al., 2015; Gao and Carson, 2016). Interestingly, mechanical stimulation via stretch also activates these signaling pathways in myotubes and are linked to skeletal muscle growth (Kumar et al., 2002; You et al., 2012; Goodman, 2019; Lin and Liu, 2019; Moustogiannis et al., 2020). Stretched myotubes treated with LLC CM exhibit blunted protein synthesis signaling and responsiveness to mechanical stimuli (Gao and Carson, 2016). Intracellular mechanical signaling can be initiated from the extracellular environment via transmembrane proteins such as integrins (Carson and Wei, 2000). Specifically, the muscle-specific integrin β1D isoform is essential for myoblast differentiation and, upon activation, initiates intracellular signaling to regulate muscle growth (Boppart and Mahmassani, 2019). Chronic stretch for 24 h increased integrin β1D protein expression in both GM and C26 conditions, which may have important implications for the transduction of mechanical and growth-related signals during cachexia. In addition to p38 MAPK and ERK1/2, mechanical stimuli can modulate the JNK pathway (Pereira et al., 2011). Acute stretch did not significantly alter JNK phosphorylation. However, studies often report rapid increases within 5–10 min, compared with the 4-h stretch used in our study (Martineau and Gardiner, 2002; Lessard et al., 2018). C26 CM did not suppress the stretch activation of p38 and ERK1/2 phosphorylation, and there was an additive effect of stretched and C26 CM on ERK1/2 phosphorylation. Therefore, it is interesting to speculate that different tumor types (i.e., LLC versus C26) may differentially alter the responsiveness to mechanical signaling. C26 CM and stretch did not alter rpS6 phosphorylation, a downstream target of mTORC1. However, the lack of observed changes could be in part due to a high growth factor environment. The induction of E3 ligases Atrogin-1/MAFbx and MuRF-1 is often associated with cancer-induced muscle wasting *in vitro* and preclinical models (Bodine and Baehr, 2014; Rom and Reznick, 2016). Studies report phenotypic differences in myotube growth when plated on various substrates (Cittadella Vigodarzere and Mantero, 2014; Ostrovidov et al., 2014; Denes et al., 2019). We sought to expand on these ideas and examined myotube growth and signaling in response to tumor-derived factors on both polystyrene and silastic membranes. In both culture conditions, C26 factors suppressed myotube growth and MyHC isoform protein expression. In myotubes differentiated on a rigid polystyrene substratum, C26 media reduced protein synthesis and increased Atrogin-1 and MuRF-1 protein expression. Surprisingly, C26 tumor-derived factors did not induce either Atrogin-1 or MuRF-1 protein expression in myotubes grown on silastic membranes. However, other signaling pathways and proteins may be involved in the growth suppression by C26 tumor-derived factors. These pathways include calpain and caspase activation, impaired protein synthesis, inflammatory cytokine-induced glucocorticoid release, and forkhead transcription factor activation (Fearon et al., 2012; Silva et al., 2015; Yamada et al., 2020). Notably, C26 CM induction of p38 MAPK and CCAAT/enhancer binding protein beta (C/EBPβ) signaling pathway can induce muscle atrophy (Sin et al., 2019). We observed a stretch suppression of E3 ligase expression, which has been previously reported (Moustogiannis et al., 2020), along with suppressing the LC3B II/I ratio. LC3B II protein levels are increased with cancer, and reduced levels suggest either reduced autophagosome formation or increased autophagosome turnover (Mizushima and Yoshimori, 2007; Aversa et al., 2016; Pettersen et al., 2017). These reductions could, in part, be mediating the protective effects of stretch on MyHC-Fast protein expression. However, mechanical stimulation can activate other pathways such as myokine release to counteract the adverse impact on myotubes treated with tumor-derived media (Baccam et al., 2019).

Skeletal muscle MyHC is a motor protein of thick filaments and the most abundant protein in skeletal muscle, and its expression is crucial in cross-bridge formation and force generation (Weiss et al., 1999; Deshmukh et al., 2015). MyHC expression is also an indicator of C2C12 myotube terminal differentiation (Silberstein et al., 1986; Brown et al., 2012), and stretch effects on the regulation of MyHC expression have been limited to this differentiation role (Wang et al., 2020). Our results suggest that stretch can rescue C26 suppression of MyHC-Fast protein expression independent of changes in MyHC isoform mRNA expression. Stretch did not alter MyHC-Slow protein expression compared with unstretched C26 + GM-treated myotubes. This isoform-specific response has implications for adult skeletal muscle since muscle fibers are heterogeneous, including MyHC fast (type IIA), intermediate/mixed (type IIX or IIB), and slow (type I) fibers (Miller and Stockdale, 1986; Ostrovidov et al., 2014). Over the course of differentiation, C2C12 myotubes display a shift in MyHC mRNA isoform expression. Specifically, type I is predominant early but declines between days 4 and 6 of differentiation, while type II isoforms (IIA, IIX, and IIB) significantly increase starting at 4 days post-differentiation (Brown et al., 2012). In the current study, myotubes were analyzed 7 days post-differentiation. Therefore, it is interesting to speculate that the temporal pattern of MyHC mRNA expression may factor into observed responses relating to the stretch rescue of MyHC-Fast and not slow isoforms. The specific phenotypic composition of fast and slow muscle MyHC isoforms determines fatigue resistance and contraction velocity (Ostrovidov et al., 2014). MyHC isoform expression is regulated at several levels, including the calcineurin/NFAT signaling pathway, MEF2C, and various microRNAs (Swoap et al., 2000; Cheng et al., 2018; Xu et al., 2018). How stretch impacts these potential control mechanisms for fast and slow isoform protein expression warrants further investigation. MEF2 transcription factors have critical roles in muscle cell differentiation and myofibers’ structural integrity. Specifically, MEF2C can control MyHC IIA expression (Allen and Leinwand, 2002; Potthoff et al., 2007), and cachexia decreases muscle MEF2C expression in C26 tumor-bearing mice (Shum et al., 2012). We provide evidence that C26-derived factors can directly suppress myotube MEF2C and MyHC IIA mRNA expression. Interestingly, in line with published *in vivo* reports (Diffee et al., 2002; Roberts et al., 2013), C26 cachexia resulted in a shift in myosin mRNA expression to have greater type IIB, while types IIA and I were reduced. Reductions in myotube contractile elements after C26 exposure seem to be specific to myosin, as skeletal α-actin mRNA is unchanged. Myoblast proliferation and fusion contribute to stretch-induced *de novo* synthesis of myofibers, which also involves inducing myogenic transcription factors (e.g., myogenin and MyoD) (Carson and Booth, 1998). Dynamic stretch in the presence of tumor-derived factors can promote myoblast fusion into nascent myotubes (Baccam et al., 2019). High growth factor serum can activate myogenic transcription factors and promote myoblast proliferation (Lawson and Purslow, 2000). While we report no effect of CM or stretch on myogenin and MyoD protein expression, stretch increased the myogenin-to-MyoD protein expression ratio. These results suggest that stretch-related growth effects can involve increased myogenic differentiation. To this end, a dynamic myotube stretch does increase the fusion index in hypertrophying myotubes (Baccam et al., 2019). Further research is warranted to examine the interaction of mechanical signaling and myogenic regulator factors to rescue tumor-derived atrophy.

In summary, we examined the effects of mechanical signaling induced by a chronic passive stretch on growth and MyHC expression in C2C12 myotubes after incubation with C26 CM. Chronic stretch administered after 24 h of C26 CM exposure provided a growth stimulus and rescued MyHC-Fast protein expression independent of mRNA expression changes. These benefits were associated with the stretch activation of ERK1/2 phosphorylation, muscle-specific E3 ubiquitin ligase suppression, LC3B II/I ratio suppression, increased integrin β1D protein expression, and increased myogenin-to-MyoD ratio. However, we must consider that mechanical signaling can activate other signaling pathways, which could also impact tumor-derived factors’ suppression of myotube growth. The impact of mechanical signaling on MyHC-Fast expression may significantly preserve or restore muscle function and mass during cancer. Investigations to define how mechanical signaling can produce this effect are highly justified. Preclinical and clinical examinations of muscle stimulation via passive movement (e.g., stretch) are well tolerated, can promote muscle growth and functional adaptations, and have clear potential to improve patient health (Carson et al., 1995; De Deyne, 2001; Deng and Cassileth, 2014; Berrueta et al., 2018). Our results suggest that mechanical stimuli can positively impact myotube growth in the presence of cachectic tumor-derived factors and a high serum environment. Understanding how mechanical signaling pathways can be successfully harnessed to benefit skeletal muscle in a catabolic cancer environment has a high potential for future cachexia therapy.

## Data Availability Statement

The original contributions presented in the study are included in the article/supplementary material, further inquiries can be directed to the corresponding author/s.

## Author Contributions

JC conceived and designed the study. JH performed the experiments. JH, BC-F, RP, and JC contributed to the statistical analysis, figure set preparation, and interpretation of the data. JH, BC-F, and JC contributed to the writing and editing of the manuscript. All authors reviewed the results and approved the final version of the manuscript.

## Conflict of Interest

The authors declare that the research was conducted in the absence of any commercial or financial relationships that could be construed as a potential conflict of interest.

## Publisher’s Note

All claims expressed in this article are solely those of the authors and do not necessarily represent those of their affiliated organizations, or those of the publisher, the editors and the reviewers. Any product that may be evaluated in this article, or claim that may be made by its manufacturer, is not guaranteed or endorsed by the publisher.
